# Dimethyl (1,1′-binaphthyl-2,2′-di­oxy)diacetate

**DOI:** 10.1107/S1600536809010824

**Published:** 2009-03-28

**Authors:** Asra Mustafa, Muhammad Raza Shah, Maimoona Khatoon, Seik Weng Ng

**Affiliations:** a HEJ Research Institute of Chemistry, International Center for Chemical and Biological Sciences, University of Karachi, Karachi 75270, Pakistan; b Department of Chemistry, University of Malaya, 50603 Kuala Lumpur, Malaysia

## Abstract

The two naphthyl fused-ring systems in the title compound, C_22_H_26_O_6_, are aligned at 86.7 (1)°. Weak inter­molecular C—H⋯O hydrogen bonding is present in the crystal structure.

## Related literature

For the crystal structure of the parent carboxylic acid, see: Wu *et al.* (2007 ▶).
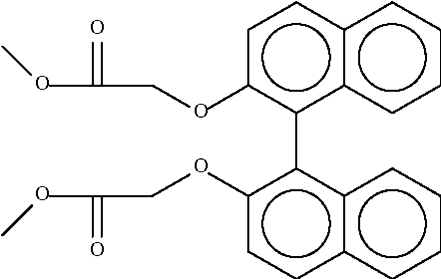


## Experimental

### 

#### Crystal data

C_26_H_22_O_6_
*M* *_r_* = 430.44
Orthorhombic, 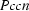

*a* = 17.1288 (5) Å
*b* = 29.439 (1) Å
*c* = 8.3518 (3) Å
*V* = 4211.5 (3) Å^3^
*Z* = 8
Mo *K*α radiation
μ = 0.10 mm^−1^
*T* = 123 K
0.27 × 0.12 × 0.05 mm

#### Data collection

Bruker SMART APEX diffractometer
Absorption correction: none
17329 measured reflections
3700 independent reflections
2613 reflections with *I* > 2σ(*I*)
*R*_int_ = 0.069

#### Refinement

*R*[*F* ^2^ > 2σ(*F* ^2^)] = 0.085
*wR*(*F* ^2^) = 0.195
*S* = 1.23
3700 reflections
244 parameters
H-atom parameters constrained
Δρ_max_ = 0.57 e Å^−3^
Δρ_min_ = −0.50 e Å^−3^

### 

Data collection: *APEX2* (Bruker, 2008 ▶); cell refinement: *SAINT* (Bruker, 2008 ▶); data reduction: *SAINT*; program(s) used to solve structure: *SHELXS97* (Sheldrick, 2008 ▶); program(s) used to refine structure: *SHELXL97* (Sheldrick, 2008 ▶); molecular graphics: *X-SEED* (Barbour, 2001 ▶); software used to prepare material for publication: *publCIF* (Westrip, 2009 ▶).

## Supplementary Material

Crystal structure: contains datablocks global, I. DOI: 10.1107/S1600536809010824/xu2498sup1.cif

Structure factors: contains datablocks I. DOI: 10.1107/S1600536809010824/xu2498Isup2.hkl

Additional supplementary materials:  crystallographic information; 3D view; checkCIF report

## Figures and Tables

**Table 1:** Hydrogen-bond geometry (Å, °)

*D*—H⋯*A*	*D*—H	H⋯*A*	*D*⋯*A*	*D*—H⋯*A*
C3—H3⋯O6^i^	0.95	2.38	3.330 (3)	174
C21—H21*A*⋯O6^i^	0.99	2.48	3.326 (5)	143
C26—H26*B*⋯O1^ii^	0.98	2.46	3.338 (6)	149

Symmetry codes: (i) 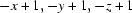; (ii) 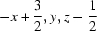.

**Fig. 1.**
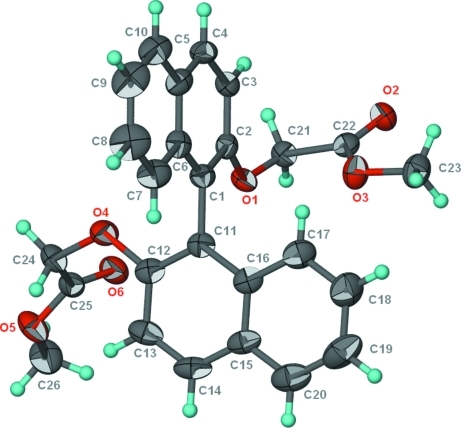
Thermal ellipsoid plot (Barbour, 2001) plot of C26H22O6 at the 70% probability level; hydrogen atoms are drawn as spheres of arbitrary radius.

## supplementary crystallographic information

### Experimental

Potassium carbonate (0.55 g, 4 mmol) and 1,1'-binaphthyl-2,2'-diol (0.29 mg, 1 mmol) in acetone (10 ml) were stirred for 15 minutes. Methyl 2-chloroacetate (0.54 g, 10 mmol) was added and the mixture was stirred at 323 K for 24twenty-four hours. The solvent was removed and the residue was dissolved in a mixture of water (25 ml) and dichloromethane (25 ml). The two phases were separated and the aqueous layer was extracted with dichloromethane. The combined organic phases were dried and the solvent evaporated. The residue was dissolved recrystallized from dichloromethane (0.33 g, 80% yield).

### Refinement

The crystal did not diffract strongly. To lower the observeds:parameters ratio, the fused rings were refined as rigid naphthalenes of 1.39 Å sides.

Carbon-bound H-atoms were placed in calculated positions (C—H 0.93 to 0.99 Å) and were included in the refinement in the riding model approximation, with U_iso_(H) set to 1.2 to 1.5U_eq_(C).

### Crystal data

**Table:**

C_26_H_22_O_6_	*F*(000) = 1808
*M**_r_* = 430.44	*D*_x_ = 1.358 Mg m^−^^3^
Orthorhombic, *P**c**c**n*	Mo *K*α radiation, λ = 0.71073 Å
Hall symbol: -P 2ab 2ac	Cell parameters from 1966 reflections
*a* = 17.1288 (5) Å	θ = 2.3–21.8°
*b* = 29.439 (1) Å	µ = 0.10 mm^−^^1^
*c* = 8.3518 (3) Å	*T* = 123 K
*V* = 4211.5 (3) Å^3^	Prism, colorless
*Z* = 8	0.27 × 0.12 × 0.05 mm

### Data collection

**Table:**

Bruker SMART APEX diffractometer	2613 reflections with *I* > 2σ(*I*)
Radiation source: fine-focus sealed tube	*R*_int_ = 0.069
graphite	θ_max_ = 25.0°, θ_min_ = 1.4°
ω scans	*h* = −19→20
17329 measured reflections	*k* = −35→34
3700 independent reflections	*l* = −9→9

### Refinement

**Table:**

Refinement on *F*^2^	Secondary atom site location: difference Fourier map
Least-squares matrix: full	Hydrogen site location: inferred from neighbouring sites
*R*[*F*^2^ > 2σ(*F*^2^)] = 0.085	H-atom parameters constrained
*wR*(*F*^2^) = 0.195	*w* = 1/[σ^2^(*F*_o_^2^) + (0.05*P*)^2^ + 9*P*] where *P* = (*F*_o_^2^ + 2*F*_c_^2^)/3
*S* = 1.23	(Δ/σ)_max_ = 0.001
3700 reflections	Δρ_max_ = 0.57 e Å^−^^3^
244 parameters	Δρ_min_ = −0.50 e Å^−^^3^
0 restraints	Extinction correction: *SHELXL97* (Sheldrick, 2008), Fc^*^=kFc[1+0.001xFc^2^λ^3^/sin(2θ)]^-1/4^
Primary atom site location: structure-invariant direct methods	Extinction coefficient: 0.0039 (6)

### Fractional atomic coordinates and isotropic or equivalent isotropic displacement parameters (Å^2^)

**Table:**

	*x*	*y*	*z*	*U*_iso_*/*U*_eq_	
O1	0.49537 (14)	0.44569 (9)	0.6224 (3)	0.0253 (7)	
O2	0.41229 (16)	0.50497 (10)	0.9571 (4)	0.0349 (8)	
O3	0.43431 (17)	0.43116 (9)	0.9055 (4)	0.0334 (7)	
O4	0.56053 (15)	0.38327 (10)	0.2796 (4)	0.0331 (7)	
O5	0.75115 (16)	0.41527 (10)	0.1635 (4)	0.0367 (8)	
O6	0.67299 (16)	0.44878 (10)	0.3437 (4)	0.0351 (8)	
C1	0.44243 (9)	0.38011 (6)	0.4970 (3)	0.0247 (9)	
C2	0.43217 (11)	0.42447 (6)	0.5501 (3)	0.0232 (9)	
C3	0.36353 (12)	0.44760 (5)	0.5150 (3)	0.0260 (9)	
H3	0.3565	0.4779	0.5512	0.031*	
C4	0.30515 (10)	0.42638 (5)	0.4268 (3)	0.0293 (10)	
H4	0.2582	0.4422	0.4028	0.035*	
C5	0.31541 (8)	0.38203 (5)	0.3738 (2)	0.0289 (10)	
C6	0.38405 (8)	0.35890 (5)	0.4089 (2)	0.0267 (9)	
C7	0.39431 (11)	0.31454 (5)	0.3558 (3)	0.0370 (11)	
H7	0.4412	0.2987	0.3798	0.044*	
C8	0.33593 (14)	0.29333 (6)	0.2677 (3)	0.0478 (13)	
H8	0.3429	0.2630	0.2314	0.057*	
C9	0.26729 (13)	0.31646 (7)	0.2326 (3)	0.0453 (13)	
H9	0.2274	0.3020	0.1723	0.054*	
C10	0.25703 (10)	0.36081 (7)	0.2856 (3)	0.0366 (11)	
H10	0.2101	0.3766	0.2616	0.044*	
C11	0.52100 (10)	0.35656 (7)	0.5345 (2)	0.0251 (9)	
C12	0.57926 (12)	0.36020 (7)	0.4193 (2)	0.0278 (10)	
C13	0.65175 (11)	0.34032 (8)	0.4455 (2)	0.0334 (11)	
H13	0.6916	0.3428	0.3668	0.040*	
C14	0.66599 (9)	0.31681 (7)	0.5869 (3)	0.0342 (11)	
H14	0.7155	0.3032	0.6048	0.041*	
C15	0.60774 (9)	0.31317 (5)	0.7020 (2)	0.0304 (10)	
C16	0.53524 (8)	0.33305 (5)	0.6758 (2)	0.0281 (10)	
C17	0.47698 (10)	0.32941 (8)	0.7910 (2)	0.0365 (11)	
H17	0.4274	0.3430	0.7731	0.044*	
C18	0.49122 (14)	0.30589 (8)	0.9323 (2)	0.0446 (13)	
H18	0.4514	0.3034	1.0110	0.054*	
C19	0.56372 (15)	0.28602 (8)	0.9585 (2)	0.0493 (14)	
H19	0.5734	0.2699	1.0551	0.059*	
C20	0.62197 (12)	0.28966 (7)	0.8434 (2)	0.0426 (12)	
H20	0.6715	0.2761	0.8613	0.051*	
C21	0.4810 (2)	0.48465 (13)	0.7182 (5)	0.0251 (9)	
H21A	0.4502	0.5067	0.6547	0.030*	
H21B	0.5316	0.4991	0.7444	0.030*	
C22	0.4379 (2)	0.47498 (14)	0.8724 (5)	0.0277 (10)	
C23	0.4005 (3)	0.41876 (16)	1.0604 (5)	0.0378 (11)	
H23A	0.3838	0.3869	1.0577	0.057*	
H23B	0.3553	0.4382	1.0825	0.057*	
H23C	0.4397	0.4229	1.1447	0.057*	
C24	0.6217 (2)	0.39136 (15)	0.1669 (5)	0.0312 (10)	
H24A	0.5997	0.4056	0.0694	0.037*	
H24B	0.6457	0.3621	0.1358	0.037*	
C25	0.6833 (2)	0.42214 (14)	0.2373 (5)	0.0279 (10)	
C26	0.8161 (3)	0.44240 (17)	0.2228 (6)	0.0441 (13)	
H26A	0.8058	0.4746	0.2020	0.066*	
H26B	0.8642	0.4333	0.1681	0.066*	
H26C	0.8219	0.4376	0.3383	0.066*	

### Atomic displacement parameters (Å^2^)

**Table:**

	*U*^11^	*U*^22^	*U*^33^	*U*^12^	*U*^13^	*U*^23^
O1	0.0193 (13)	0.0271 (15)	0.0295 (17)	0.0006 (11)	0.0016 (12)	−0.0053 (13)
O2	0.0353 (16)	0.0398 (17)	0.0297 (18)	0.0082 (14)	0.0038 (14)	−0.0048 (15)
O3	0.0429 (17)	0.0302 (16)	0.0271 (17)	−0.0061 (13)	0.0034 (14)	0.0016 (14)
O4	0.0216 (14)	0.0433 (18)	0.0346 (18)	0.0013 (12)	0.0041 (13)	0.0074 (15)
O5	0.0238 (14)	0.0423 (17)	0.044 (2)	−0.0057 (13)	0.0102 (14)	−0.0114 (16)
O6	0.0338 (16)	0.0358 (17)	0.0356 (18)	0.0062 (13)	0.0020 (14)	−0.0068 (16)
C1	0.022 (2)	0.025 (2)	0.027 (2)	0.0001 (16)	0.0015 (17)	0.0037 (19)
C2	0.0209 (19)	0.028 (2)	0.020 (2)	−0.0054 (16)	0.0017 (17)	0.0017 (18)
C3	0.023 (2)	0.029 (2)	0.025 (2)	0.0010 (17)	0.0026 (17)	0.0002 (19)
C4	0.020 (2)	0.036 (2)	0.031 (2)	0.0028 (17)	0.0018 (18)	0.009 (2)
C5	0.022 (2)	0.036 (2)	0.029 (2)	−0.0070 (17)	−0.0004 (18)	0.009 (2)
C6	0.026 (2)	0.027 (2)	0.027 (2)	−0.0061 (16)	0.0037 (18)	0.0028 (19)
C7	0.031 (2)	0.035 (2)	0.045 (3)	−0.0047 (19)	−0.005 (2)	0.002 (2)
C8	0.045 (3)	0.033 (3)	0.065 (4)	−0.015 (2)	−0.005 (3)	0.000 (3)
C9	0.038 (3)	0.045 (3)	0.053 (3)	−0.021 (2)	−0.011 (2)	0.004 (3)
C10	0.025 (2)	0.043 (3)	0.043 (3)	−0.0110 (19)	−0.002 (2)	0.012 (2)
C11	0.023 (2)	0.022 (2)	0.030 (2)	−0.0037 (16)	−0.0040 (18)	−0.0036 (19)
C12	0.025 (2)	0.024 (2)	0.034 (3)	−0.0029 (16)	−0.0050 (19)	−0.001 (2)
C13	0.022 (2)	0.029 (2)	0.049 (3)	−0.0001 (17)	0.000 (2)	−0.006 (2)
C14	0.025 (2)	0.021 (2)	0.056 (3)	0.0032 (17)	−0.011 (2)	−0.003 (2)
C15	0.031 (2)	0.020 (2)	0.041 (3)	0.0019 (17)	−0.012 (2)	−0.003 (2)
C16	0.029 (2)	0.021 (2)	0.034 (3)	−0.0037 (16)	−0.0042 (19)	0.000 (2)
C17	0.040 (3)	0.028 (2)	0.042 (3)	0.0017 (19)	−0.003 (2)	0.005 (2)
C18	0.056 (3)	0.035 (3)	0.043 (3)	0.002 (2)	0.002 (2)	0.011 (2)
C19	0.069 (4)	0.028 (2)	0.051 (3)	0.003 (2)	−0.020 (3)	0.008 (2)
C20	0.043 (3)	0.027 (2)	0.058 (3)	0.005 (2)	−0.015 (3)	−0.005 (2)
C21	0.026 (2)	0.022 (2)	0.027 (2)	−0.0015 (16)	0.0001 (18)	−0.0026 (18)
C22	0.023 (2)	0.034 (2)	0.026 (2)	0.0006 (17)	−0.0042 (18)	−0.001 (2)
C23	0.039 (3)	0.044 (3)	0.030 (3)	−0.010 (2)	0.000 (2)	0.006 (2)
C24	0.026 (2)	0.036 (2)	0.032 (3)	0.0001 (17)	0.0076 (19)	−0.002 (2)
C25	0.030 (2)	0.025 (2)	0.029 (2)	0.0056 (17)	0.0039 (19)	0.003 (2)
C26	0.032 (2)	0.053 (3)	0.046 (3)	−0.011 (2)	0.002 (2)	−0.007 (3)

### Geometric parameters (Å, °)

**Table:**

O1—C2	1.388 (3)	C11—C16	1.3900
O1—C21	1.420 (5)	C12—C13	1.3900
O2—C22	1.213 (5)	C13—C14	1.3900
O3—C22	1.321 (5)	C13—H13	0.9500
O3—C23	1.464 (5)	C14—C15	1.3900
O4—C12	1.388 (3)	C14—H14	0.9500
O4—C24	1.428 (5)	C15—C16	1.3900
O5—C25	1.330 (5)	C15—C20	1.3900
O5—C26	1.456 (5)	C16—C17	1.3900
O6—C25	1.199 (5)	C17—C18	1.3900
C1—C2	1.3900	C17—H17	0.9500
C1—C6	1.3900	C18—C19	1.3900
C2—C3	1.3900	C18—H18	0.9500
C3—C4	1.3900	C19—C20	1.3900
C3—H3	0.9500	C19—H19	0.9500
C4—C5	1.3900	C20—H20	0.9500
C4—H4	0.9500	C21—C22	1.511 (6)
C5—C6	1.3900	C21—H21A	0.9900
C5—C10	1.3900	C21—H21B	0.9900
C6—C7	1.3900	C23—H23A	0.9800
C7—C8	1.3900	C23—H23B	0.9800
C7—H7	0.9500	C23—H23C	0.9800
C8—C9	1.3900	C24—C25	1.511 (6)
C8—H8	0.9500	C24—H24A	0.9900
C9—C10	1.3900	C24—H24B	0.9900
C9—H9	0.9500	C26—H26A	0.9800
C10—H10	0.9500	C26—H26B	0.9800
C11—C12	1.3900	C26—H26C	0.9800
			
C2—O1—C21	118.3 (2)	C17—C16—C15	120.0
C22—O3—C23	116.5 (3)	C17—C16—C11	120.0
C12—O4—C24	117.8 (3)	C15—C16—C11	120.0
C25—O5—C26	115.2 (3)	C16—C17—C18	120.0
C2—C1—C6	120.0	C16—C17—H17	120.0
O1—C2—C1	117.57 (16)	C18—C17—H17	120.0
O1—C2—C3	122.06 (16)	C19—C18—C17	120.0
C1—C2—C3	120.0	C19—C18—H18	120.0
C4—C3—C2	120.0	C17—C18—H18	120.0
C4—C3—H3	120.0	C18—C19—C20	120.0
C2—C3—H3	120.0	C18—C19—H19	120.0
C3—C4—C5	120.0	C20—C19—H19	120.0
C3—C4—H4	120.0	C19—C20—C15	120.0
C5—C4—H4	120.0	C19—C20—H20	120.0
C6—C5—C4	120.0	C15—C20—H20	120.0
C6—C5—C10	120.0	O1—C21—C22	114.4 (3)
C4—C5—C10	120.0	O1—C21—H21A	108.7
C7—C6—C5	120.0	C22—C21—H21A	108.7
C7—C6—C1	120.0	O1—C21—H21B	108.7
C5—C6—C1	120.0	C22—C21—H21B	108.7
C8—C7—C6	120.0	H21A—C21—H21B	107.6
C8—C7—H7	120.0	O2—C22—O3	124.9 (4)
C6—C7—H7	120.0	O2—C22—C21	122.4 (4)
C7—C8—C9	120.0	O3—C22—C21	112.6 (3)
C7—C8—H8	120.0	O3—C23—H23A	109.5
C9—C8—H8	120.0	O3—C23—H23B	109.5
C10—C9—C8	120.0	H23A—C23—H23B	109.5
C10—C9—H9	120.0	O3—C23—H23C	109.5
C8—C9—H9	120.0	H23A—C23—H23C	109.5
C9—C10—C5	120.0	H23B—C23—H23C	109.5
C9—C10—H10	120.0	O4—C24—C25	110.9 (4)
C5—C10—H10	120.0	O4—C24—H24A	109.5
C12—C11—C16	120.0	C25—C24—H24A	109.5
O4—C12—C13	123.01 (16)	O4—C24—H24B	109.5
O4—C12—C11	116.98 (16)	C25—C24—H24B	109.5
C13—C12—C11	120.0	H24A—C24—H24B	108.0
C12—C13—C14	120.0	O6—C25—O5	124.9 (4)
C12—C13—H13	120.0	O6—C25—C24	125.3 (4)
C14—C13—H13	120.0	O5—C25—C24	109.8 (4)
C15—C14—C13	120.0	O5—C26—H26A	109.5
C15—C14—H14	120.0	O5—C26—H26B	109.5
C13—C14—H14	120.0	H26A—C26—H26B	109.5
C14—C15—C16	120.0	O5—C26—H26C	109.5
C14—C15—C20	120.0	H26A—C26—H26C	109.5
C16—C15—C20	120.0	H26B—C26—H26C	109.5
			
C21—O1—C2—C1	−161.7 (2)	C11—C12—C13—C14	0.0
C21—O1—C2—C3	25.3 (4)	C12—C13—C14—C15	0.0
C6—C1—C2—O1	−173.2 (2)	C13—C14—C15—C16	0.0
C6—C1—C2—C3	0.0	C13—C14—C15—C20	180.0
O1—C2—C3—C4	172.9 (2)	C14—C15—C16—C17	180.0
C1—C2—C3—C4	0.0	C20—C15—C16—C17	0.0
C2—C3—C4—C5	0.0	C14—C15—C16—C11	0.0
C3—C4—C5—C6	0.0	C20—C15—C16—C11	180.0
C3—C4—C5—C10	180.0	C12—C11—C16—C17	180.0
C4—C5—C6—C7	180.0	C12—C11—C16—C15	0.0
C10—C5—C6—C7	0.0	C15—C16—C17—C18	0.0
C4—C5—C6—C1	0.0	C11—C16—C17—C18	180.0
C10—C5—C6—C1	180.0	C16—C17—C18—C19	0.0
C2—C1—C6—C7	180.0	C17—C18—C19—C20	0.0
C2—C1—C6—C5	0.0	C18—C19—C20—C15	0.0
C5—C6—C7—C8	0.0	C14—C15—C20—C19	180.0
C1—C6—C7—C8	180.0	C16—C15—C20—C19	0.0
C6—C7—C8—C9	0.0	C2—O1—C21—C22	68.8 (4)
C7—C8—C9—C10	0.0	C23—O3—C22—O2	−3.5 (6)
C8—C9—C10—C5	0.0	C23—O3—C22—C21	174.1 (3)
C6—C5—C10—C9	0.0	O1—C21—C22—O2	−170.2 (4)
C4—C5—C10—C9	180.0	O1—C21—C22—O3	12.1 (5)
C24—O4—C12—C13	−7.6 (4)	C12—O4—C24—C25	−64.1 (4)
C24—O4—C12—C11	173.8 (2)	C26—O5—C25—O6	1.1 (6)
C16—C11—C12—O4	178.6 (2)	C26—O5—C25—C24	−178.8 (4)
C16—C11—C12—C13	0.0	O4—C24—C25—O6	−24.5 (6)
O4—C12—C13—C14	−178.5 (2)	O4—C24—C25—O5	155.4 (3)

### Hydrogen-bond geometry (Å, °)

**Table:**

*D*—H···*A*	*D*—H	H···*A*	*D*···*A*	*D*—H···*A*
C3—H3···O6^i^	0.95	2.38	3.330 (3)	174
C21—H21A···O6^i^	0.99	2.48	3.326 (5)	143
C26—H26B···O1^ii^	0.98	2.46	3.338 (6)	149

Symmetry codes: (i) −*x*+1, −*y*+1, −*z*+1; (ii) −*x*+3/2, *y*, *z*−1/2.
